# Focus, vigilance, resilience: towards stronger infectious disease surveillance, threat detection and response in the EU/EEA

**DOI:** 10.2807/1560-7917.ES.2024.29.34.2400066

**Published:** 2024-08-22

**Authors:** Phillip Zucs, Julien Beauté, Daniel Palm, Gianfranco Spiteri

**Affiliations:** 1 European Centre for Disease Prevention and Control (ECDC), Stockholm, Sweden

**Keywords:** Europe, EU/EEA, infectious diseases, surveillance, strategic framework, sentinel surveillance, molecular methods

## Abstract

This perspective summarises and explains the long-term surveillance framework 2021–2027 for infectious diseases in the European Union/European Economic Area (EU/EEA) published in April 2023. It shows how shortcomings in the areas of public health focus, vigilance and resilience will be addressed through specific strategies in the coming years and how these strategies will lead to stronger surveillance systems for early detection and monitoring of public health threats as well as informing their effective prevention and control. A sharper public health focus is expected from a more targeted list of notifiable diseases, strictly public-health-objective-driven surveillance standards, and consequently, leaner surveillance systems. Vigilance should improve through mandatory event reporting, more automated epidemic intelligence processing and increased use of genomic surveillance. Finally, EU/EEA surveillance systems should become more resilient by modernising the underlying information technology infrastructure, expanding the influenza sentinel surveillance system to other respiratory viruses for better pandemic preparedness, and increasingly exploiting potentially more robust alternative data sources, such as electronic health records and wastewater surveillance. Continued close collaboration across EU/EEA countries will be key to ensuring the full implementation of this surveillance framework and more effective disease prevention and control.

## Background

### Infectious disease surveillance structures and strategic framework

Infectious disease surveillance at European Union/European Economic Area (EU/EEA) level is coordinated by the European Centre for Disease Prevention and Control (ECDC) and consists of two complementary pillars – indicator-based surveillance and event-based surveillance (Table). Indicator-based surveillance is the continuous or periodic collection, analysis and dissemination of structured data on cases or clinical isolates of pathogens to inform public health decision-making. It is carried out by disease-specific networks of nationally nominated experts who report national surveillance data to ECDC, where EU/EEA data are then compiled, validated, analysed and disseminated. Event-based surveillance is the ad hoc collection, monitoring, assessment and interpretation of unstructured information relating to potential public health threats. At EU/EEA level, this is carried out by nationally nominated experts reporting public health events with a possible cross-border dimension to dedicated communication platforms operated by the European Commission and ECDC. This is complemented by ECDC experts screening publicly available global online sources and restricted platforms several times a day for epidemic intelligence.

**Table:** Interplay of indicator-based and event-based surveillance at EU/EEA level

Objective	Indicator-based surveillance		Event-based surveillance	
	Epidemiological	Genomic	EU/EEA country reporting	ECDC epidemic intelligence
Monitor: − Disease burden − Strain distribution − Long-term trend − Risk groups − Prevention and control programmes	X	X	NA	NA
Threat detection, monitoring and assessment	X	X	X	X
Global threat monitoring and assessment	NA	NA	NA	X

ECDC: European Centre for Disease Prevention and Control; EEA: European Economic Area; EU: European Union; NA: not applicable.

On 4 April 2023, after extensive consultations between EU/EEA countries, the ECDC Advisory Forum (including the European Commission and World Health Organization (WHO)) and the ECDC Management Board, ECDC published a strategic framework for infectious disease surveillance in the EU/EEA [1]. This framework covers the years 2021 to 2027 and defines the strategic actions, targets and milestones to be accomplished in collaboration with the European Commission and the 30 EU/EEA countries. To a large part, it aims to eliminate shortcomings that have hampered infectious disease surveillance, threat detection and response at EU/EEA level for years.

### Success stories: some examples

There are several examples of EU/EEA infectious disease surveillance systems that have been effective in enabling timely threat detection and response, or long-term monitoring and adjustment of public health programmes. Within less than a week from the first cases reported by an EU country, ECDC had compiled the data relating to 67 confirmed cases of mpox in nine EU countries and published its first risk assessment [2]. The near real-time surveillance of travel-associated Legionnaires’ disease has led to the detection and control of numerous clusters linked to hotels and other commercial tourist accommodations [3]. The weekly reporting of locally acquired cases of West Nile virus infection during the transmission season has helped identify areas of risk in the EU/EEA and their neighbouring countries and thus made blood donations safer [4]. Routine collection of whole genome sequencing (WGS) data has facilitated the detection and investigation of multi-country food-borne outbreaks [5] and the monitoring of severe acute respiratory syndrome coronavirus 2 (SARS-CoV-2) variants of concern [6]. The point prevalence surveys of healthcare-associated infections and antimicrobial use in acute care hospitals and long-term care facilities have provided a major push towards more determined infection prevention and control in those settings [7].

### Challenges

Some features of EU/EEA surveillance, however, reveal insufficient public health focus and vigilance. The list of infectious diseases and related health issues notifiable at EU/EEA level comprises almost 60 items [8]. Each of these diseases and health issues has been under indicator-based surveillance, requiring periodic collection, validation, analysis and dissemination of structured data from all EU/EEA countries. This list, however, has never been systematically reviewed and updated based on public health priority and ability to inform prevention and control strategies. Some disease-specific lists of variables to be reported at EU/EEA level include several that are poorly completed by participating countries or not routinely analysed by ECDC. Indicator-based surveillance cycles have been mostly annual across diseases and hence too slow for any timely cross-border outbreak detection and response. Despite achievements in outbreak detection and investigation, the transition towards comprehensive and integrated surveillance for food and waterborne diseases, adopting WGS and effective sampling strategies across human, food, feed and environmental sectors, has been slow and hampered by divergent interests. Some of these challenges have been mirrored at the national level with some countries hardly contributing to EU/EEA event-based surveillance, probably partly due to a lack of capacity. Eleven of 30 EU/EEA countries, for example, did not post a single item on a potential food and waterborne disease event on the dedicated ECDC platform between July 2021 and December 2023 (EpiPulse Events, unpublished data).

The COVID-19 pandemic turned the spotlight on another important weakness of EU/EEA infectious disease surveillance: its lack of resilience. Comprehensive case-based surveillance placed an enormous reporting burden on healthcare workers who were already stretched to the limit and therefore provided delayed and often very incomplete data. Even when switching to aggregated indicator data, many EU/EEA countries could not keep up. Analyses of the effectiveness of medical countermeasures at EU/EEA level were not possible or were delayed partly due to the lack of enhanced surveillance data. Missing integration of data sources across healthcare levels impeded monitoring of severity and impact. Frequent and disparate changes in testing and social distancing policies could not be accounted for and rendered surveillance data poorly comparable between countries and over time. Some countries are still struggling to implement representative integrated sentinel surveillance systems for respiratory viral infections that can provide alerts, guide public health measures, inform vaccination strategies and ensure pandemic preparedness.

In late 2022, partly as a reaction to the COVID-19 pandemic, the EU strengthened the ECDC legal mandate and toolbox with a revised founding regulation [9] and expanded legislation on prevention and control of serious cross-border threats to health [10]. The EU/EEA long-term surveillance framework reflects these changes, and this paper highlights the most important ongoing and future developments geared to help EU/EEA infectious disease surveillance fulfil its potential and maximise its public health impact.

## Focus: more targeted indicator-based surveillance

The current list of infectious diseases and related special health issues notifiable at EU/EEA level will be revised in a secondary act complementing the new legislation on serious cross-border threats to health. Only diseases potentially requiring coordinated prevention and control (e.g. early warning, outbreak investigation, monitoring of public health programmes) will be under EU/EEA surveillance. Less common outbreak-prone diseases may only require reporting on events fulfilling specific criteria for public health relevance at EU/EEA level and hence no longer require routine transfer of national indicator-based data to ECDC and subsequent data validation and dissemination. This will allow ECDC and its disease networks to focus on disease areas where indicator-based surveillance adds the most public health value at EU/EEA level.

For each disease under EU/EEA surveillance, explicit public health action-oriented surveillance objectives will help define the most appropriate surveillance outputs. These, in turn, will determine the choice of surveillance systems, variables to be collected and reporting frequency. Aiming for the leanest possible system necessary to achieve the given surveillance objectives will result in a sharper focus on public health value and less waste of resources. However, public health prioritisation and efficiency needs will have to be carefully balanced against the growing demand for surveillance data to inform e.g. policy, capacity planning and investments, patient management, vaccine composition and vaccination strategies, or forecasting.

## Vigilance: more committed surveillance of events

Another secondary act complementing the new legislation on serious cross-border threats to health will define the minimum requirements for national surveillance data reporting to the EU/EEA level. This act will, for the first time, specify whether a disease is under indicator-based surveillance, event-based surveillance, or both. In addition, this act will list the events (linked to specific diseases or other public health emergency scenarios) to be promptly notified. This signifies a paradigm shift: event-based surveillance will become a legal obligation and, for a considerable subset of infectious diseases, the only form of surveillance at EU/EEA level. In many instances, however, this will require a continuation of national routine indicator-based surveillance as a baseline for signal detection. It also does not preclude a temporary return to supranational indicator-based reporting, should the magnitude or duration of an event so require. Therefore, while event-based surveillance will be enhanced, the legal and operational prerequisites for indicator-based surveillance will need to be maintained both at national and EU/EEA level.

In line with their strategic roadmap [11], ECDC and EU/EEA countries will continue to roll out and firmly establish WGS as the laboratory method of choice to detect and delineate genomic clusters, identify sources and help contain multi-country outbreaks as quickly as possible. The new legislation on serious cross-border threats to health specifically points out the obligation for EU/EEA countries to report molecular pathogen data, if required for detecting or investigating serious cross-border threats to health [10]. Following a One Health approach, a molecular typing system operated jointly with the European Food Safety Agency will ensure timely follow-up of public health signals from the veterinary and food sector [12].

For a number of outbreak-prone pathogens, ECDC and EU/EEA countries will set up weekly aggregate reporting of laboratory-confirmed cases at the EU/EEA level to detect and further investigate any aberrations from historical reporting baselines. Ideally, national data would be collected from existing automated laboratory information management systems, and the entire process, including data transfer to ECDC, data compilation and weekly analysis would be mostly automated to avoid any unnecessary additional workload. This surveillance system would also capture cases confirmed by methods other than advanced molecular technology. It would therefore include countries currently not able to routinely carry out WGS, which could considerably improve comprehensive early threat detection and response.

Epidemic intelligence processes at ECDC from signal detection to the production of routine outputs (e.g. ECDC Communicable Disease Threats Report) will be further automated for improved timeliness and more efficient use of human resources. Artificial intelligence tools will be developed to support signal filtering, provide contextual information on events and support their assessment. Enhanced collaboration with EU/EEA country focal points for threat detection as well as national public health institutes beyond the EU/EEA will strengthen formal and informal communication channels and facilitate rapid exchange of information in times of public health crisis.

## Resilience: more robust systems and data sources

The European Centre for Disease Prevention and Control is building a new online surveillance platform called EpiPulse. Accessible to users nominated by their national competent body, it will be a one-stop shop for indicator-based and event-based surveillance, enabling seamless digital data reporting, validation, exploration and extraction. To facilitate the use of genomic data, EpiPulse will offer user-friendly functionalities for their upload, analysis and integrated visualisation. Based on all these surveillance components, the platform will enable ECDC to provide EU/EEA countries with timely and comprehensive situation awareness on events. EpiPulse will be scalable to pandemic data volumes, offering both the IT processing power and automated workflows to keep the system running smoothly during public health emergencies.

Learning from one of the major surveillance shortcomings during the COVID-19 pandemic, ECDC and most EU/EEA countries have set out to fully integrate sentinel surveillance of respiratory viral infections [13]. Rather than comprehensive surveillance mirroring ever-changing testing and disease control policies, a defined sample of clinical specimens from patients presenting to sentinel primary care and hospital sites with influenza-like illness, acute respiratory infection or severe acute respiratory infection will be tested for SARS-CoV-2, influenza virus and respiratory syncytial virus. This should ensure a steady data flow and improve data comparability over time and between countries. It should also massively reduce the reporting burden for the vast majority of healthcare workers, thus ensuring the necessary focus on clinical care during the next respiratory viral pandemic.

In order to limit the reporting burden even further, ECDC and EU/EEA countries have embarked on the secondary use of electronically stored health data for routine surveillance of severe acute respiratory infection, bloodstream infections, and sexually transmitted infections. Many EU/EEA countries are still facing considerable challenges with access to, and the quality of data derived from electronic health records. Once they are more firmly established, these systems will enable automatic extraction of healthcare data from existing databases, ideally not only freeing up working time of scarce clinical and public health human resources but also improving data quality and subsequent decision-making. This may also help define standards for coding data in electronic health records for public health purposes.

Another data source that received special attention during the COVID-19 pandemic was wastewater surveillance which had previously been mostly used for tracking poliovirus [14]. Wastewater surveillance of SARS-CoV-2 and its variants has provided very useful early warning signals and trend data complementing routine surveillance while not depending on clinical testing and hence avoiding its inherent biases [15]. The European Commission and EU/EEA countries have started to establish routine wastewater surveillance of relevant pathogens across the EU/EEA. The European Centre for Disease Prevention and Control will work closely with all parties involved to integrate these data with data from existing surveillance systems for better insights into infectious disease dynamics across public health and environmental sectors.

## The foundation: capacity, standards, collaboration

### Capacity

In conjunction with its extended new mandate, ECDC has received some 70 new staff positions. A notable number of new staff profiles are in areas such as data science, data management, data visualisation, microbiology, mathematical modelling, bioinformatics and e-health. This will directly strengthen the different pillars of EU/EEA surveillance and support the implementation of the long-term surveillance framework.

Since 2021, ECDC has assisted the European Commission in implementing two major programmes to boost laboratory infrastructure (equipment and human resources) for WGS and PCR in EU/EEA countries. In the coming years, similar Commission funds will be made available to promote the digitalisation and integration of national epidemiological surveillance systems. Linked to the infrastructure investments, ECDC is offering a multidisciplinary training programme in genomic epidemiology and public health bioinformatics to further strengthen countries’ laboratory and epidemiologic capacities.

The European Centre for Disease Prevention and Control will continue to run its continuing professional development and 2-year fellowship programmes (European Programme for Intervention Epidemiology Training (EPIET) and European Programme for Public Health Microbiology Training (EUPHEM)) [16] to train current and future generations of public health epidemiologists and microbiologists. The training curricula of these programmes are regularly updated to reflect the rapidly growing importance of subdisciplines such as genomic epidemiology, mathematical modelling, data science and public health informatics. The European Centre for Disease Prevention and Control also supports infectious disease surveillance capacity building in EU enlargement countries, European Neighbourhood countries and in Africa.

### Standards

The European Centre for Disease Prevention and Control and EU/EEA countries will agree on disease- or disease-group-specific indicator-based, event-based and molecular surveillance standards to adhere or aspire to. These will be minimum requirements to be met for any given EU/EEA surveillance system to achieve its stated public health objectives. Standards will include a basic set of surveillance system parameters, such as the data sources, the variables to be collected, the frequency of reporting, but also the minimum level of completeness for some key variables and the deadlines for reporting and data dissemination [17]. The European Centre for Disease Prevention and Control will monitor and regularly report on compliance with these surveillance standards including its own obligations. Besides strengthening surveillance harmonisation and quality across EU/EEA countries, these standards also offer the opportunity to remove obsolete features at the EU/EEA level, such as long-standing variables collected, or outputs produced, without tangible public health gain.

The standard-setting in laboratory matters will be coordinated by newly designated European reference laboratories. They will coordinate methods standardisation, external quality assessments and trainings within their respective network of national reference laboratories, thus ensuring the validity and comparability of laboratory confirmations, typing and sequencing results across the EU/EEA.

### Collaboration

EU/EEA surveillance is a collective effort. Since EU/EEA countries are often facing the same public health threats from infectious diseases, they need to merge their forces for maximum protection. The European Centre for Disease Prevention and Control and EU/EEA countries are fortunate in having strong and experienced disease networks in place for whom surveillance is a major priority and who have come a long way in harmonising their surveillance systems, data and practices. Close collaboration across national borders, within these networks and beyond, will be instrumental in strengthening infectious disease surveillance, threat detection and response in the EU/EEA and accomplishing the long-term surveillance framework’s vision of “a digital data stream that provides the right information where and when it is needed to most timely and effectively fight cross-border threats to public health from infectious diseases.” [1]

## Conclusion

The long-term surveillance framework 2021–2027 for infectious diseases in the EU/EEA lays down strategies to address some of the current challenges of surveillance at EU/EEA level. The framework is ambitious, yet feasible as it builds on the solid foundation of existing surveillance structures and mechanisms as well as the long-standing collective experience of well-functioning surveillance networks. With renewed public health focus, vigilance and resilience, EU/EEA infectious disease surveillance will be better equipped to address the known threats of today and better prepared for any unknown threats emerging in the future.
